# Growing evening primroses (*Oenothera*)

**DOI:** 10.3389/fpls.2014.00038

**Published:** 2014-02-13

**Authors:** Stephan Greiner, Karin Köhl

**Affiliations:** Max Planck Institute of Molecular Plant PhysiologyPotsdam, Germany

**Keywords:** *Oenothera*, evening primrose, experimental culture, growth requirements, propagation, pest control

## Abstract

The model plant *Oenothera* has contributed significantly to the biological sciences and it dominated the early development of plant genetics, cytogenetics, and evolutionary biology. The great advantage of using *Oenothera* as a model system is a large body of genetic, cytological, morphological, and ecological information collected over more than a century. The *Oenothera* system offers a well-studied taxonomy, population structure, and ecology. Cytogenetics and formal genetics at the population level are extensively developed, providing an excellent basis to study evolutionary questions. Further, *Oenothera* is grown as an oil seed crop for the production of essential fatty acids (gamma-linoleic acid) and is considered to be a medicinal plant due to its many pharmaceutically active secondary metabolites, such as ellagitannins. Although *Oenothera* has been cultivated as a laboratory organism since the end of the 19th century, there is a substantial lack of literature dealing with modern greenhouse techniques for the genus. This review compiles an overview about the growth requirements for the genus *Oenothera*, with a special focus on its genetically best-studied subsections *Oenothera* and *Munzia*. Requirements for greenhouse, field, and agronomic cultures are presented, together with information on substrate types, pest control, as well as vegetative and seed propagation, cross pollination, harvest, and seed storage. Particular aspects like germination, bolting, and flowering induction in taxonomically diverse material are reviewed. Methods recommended are supported by ecological and experimental data. An overview of the possibilities for wide hybridization and polyploidy induction in the genus is given. Germplasm resources are referenced. In summary, a comprehensive guideline for successful laboratory cultivation of *Oenothera* species is provided.

## INTRODUCTION

The first reports on growing *Oenothera* (evening primrose, Onagraceae) coincide with the early rise of genetics. For example, pioneering work on *Oenothera* led Hugo de Vries to the formulation of his mutation theory (de Vries, 1901–1903; Nei and Nozawa, 2011), and evening primroses dominated classical cytoplasmic genetics (Chiu and Sears, 1993; Harte, 1994; Hagemann, 2010; Johnson, 2010). At the same time, the genus became a notable model to study non-Mendelian inheritance, plastome–genome co-evolution, cytoplasmic elements in plant adaptation and speciation, suppression of genome-wide recombination, chromosome translocations, and evolutionary ecology (Golczyk et al., 2008; Greiner et al., 2008; Rauwolf et al., 2008; Johnson et al., 2009b; Artz et al., 2010; Johnson, 2010; Theiss et al., 2010; Brown and Levin, 2011; Evans et al., 2011; Greiner et al., 2011; Rauwolf et al., 2011; Agrawal et al., 2012; Greiner, 2012; von Arx et al., 2012; Burton et al., 2013; Greiner and Bock, 2013). Furthermore, *Oenothera* is exploited as a crop for the production of the essential gamma-linoleic fatty acid (Deng et al., 2001; Fieldsend, 2007). Many of its secondary metabolites, such as ellagitannins (e.g., oenothein B, which suppresses tumor development), are of increasing importance (for references, see Singh et al., 2012).

Although research on *Oenothera* has been conducted for more than 120 years, there are only a few summaries available describing cultivation techniques for evening primrose as a laboratory organism. Relevant information was either published very early in the German literature, or was never published but passed on within schools of *Oenothera* workers. Most of these methods, however, only refer to field experiments and almost no reports cover modern greenhouse cultivation. This is in sharp contrast to a substantial and emerging literature describing crop management of *Oenothera* in agronomics. The aim of the present work is to summarize published and unpublished methods used by generations of *Oenothera* researchers. In addition, methods for greenhouse cultivation, developed at the Ludwig-Maximilians-University in Munich, Germany, as well as the Max Planck Institute of Molecular Plant Physiology, Potsdam–Golm, Germany, are presented. All methods are set into an ecological context and a summary of the comprehensive experimental literature supporting these methods is given.

Currently, the genus *Oenothera* consists of 145 species subdivided into 18 sections^1^. It colonizes a wide range of habitats and climate zones. Although originated in the Americas, some of its species easily adapt and the genus includes nearly cosmopolitan but also endemic taxa (Wagner et al., 2007). This results in a huge diversification, e.g., in the requirements for flower induction, which can vary even within a species (e.g., Evans et al., 2005; Johnson, 2007; and references therein). Hence, the synchronization of large numbers of *Oenothera* cultivars containing multiple genotypes is challenging. Protocols described in this work are based on approaches developed for the well-studied section *Oenothera*, that currently contains 65 species in the six subsections *Oenothera*, *Raimannia*, *Munzia*, *Candela*, *Emersonia*, and *Nutantigemma* (Wagner et al., 2007). Among them, genetically most important are subsection *Oenothera* (syn: *Euoenothera*; Dietrich et al., 1997) and species formally grouped into the sections/subsections *Raimannia*, *Renneria*, *Anogra*, and *Eu-Oenothera*. The genetically studied material of the latter groups is now included within section *Kleinia* and subsections *Munzia* and *Candela* (Dietrich, 1977; Dietrich and Wagner, 1988; Wagner et al., 2007). Extensive genetic literature exists for this material (for review, see Cleland, 1972; Harte, 1994). For subsection *Oenothera*, detailed physiological studies have facilitated the development of growth protocols, with the original methods referring to the subsections *Oenothera* and *Munzia* (de Vries, 1913; Schwemmle et al., 1938). These methods can, however, be used for the whole section *Oenothera* (cf., Stubbe and Raven, 1979a) and for material from other sections, like *Oenothera cespitosa* (section *Pachylophus*), *Oe. speciosa* (section *Hartmannia*), or *Oe. macrocarpa* (section *Megapterium*). The protocols may apply thus to most *Oenothera* species, even if specific culture recommendations given in this work largely refer to the subsection *Oenothera* and the genetic stocks of Julius Schwemmle and co-workers (subsection *Munzia*).

## GENERAL CHARACTERISTICS

**FIGURE 1:**
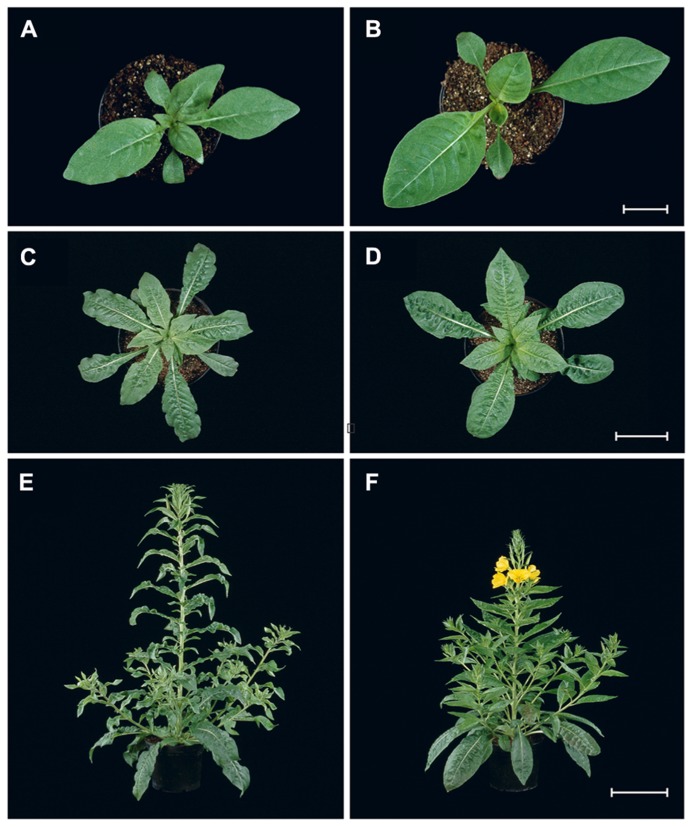
**Developmental series of plants from subsection *Oenothera* under controlled greenhouse conditions.** *Oe. elata* **(A,C,E)**, *Oe. biennis* **(B,D,F)**. Beginning of early rosette stage, 21 days after germinations **(A,B)**. Bar: 2 cm. Note variation in leaf number and size between the two species. Mature rosettes just before bolting, 42 days after germination **(C,D)**. Bar: 10 cm. Mature plants, 77 days after germination **(E,F)**. Bar: 20 cm. *Oe. biennis* already starts blooming **(F)**, whereas *Oe. elata* will flower in approximately 1 week **(E)**.

*Oenothera* is an herbaceous plant. Species of subsection *Oenothera* are facultative biennials or short-lived perennials, 40 cm*–*2.5 m (4.0 m) in size with typical heights of 1.0*–*1.5 m. Rosette size can vary between 10 and 40 cm, reaching the upper limit when cultivated (**Figures 1C,D**). Subsection *Munzia* is annual or biennial, with a huge height variation, from a few cms to 2.0 m with rosette diameters of 5*–*70 cm (Dietrich, 1977; Hall et al., 1988; Harte, 1994; Dietrich et al., 1997; Wagner et al., 2007). Many diploid species in the genus are permanent translocation heterozygotes, i.e., due to reciprocal chromosome translocations meiotic rings are formed. This leads to formation of two superlinkage groups, so called Renner complexes, each involving one complete haploid chromosome set (α and β). Specialized breeding behavior, e.g., gametophytic lethal factors, can eliminate homozygous segregants (α.α or β.β), resulting in permanent heterozygous offspring (α.β) only (Cleland, 1972). Within subsection *Oenothera*, three basic nuclear genomes (A, B, and C) occurring in homozygous (AA, BB, and CC) or stable heterozygous (AB, AC, and BC) combinations could be identified. These are associated with five basic plastome (chloroplast genome) types (I*–*V). Combinations of distinct basic nuclear genomes with basic plastome types represent an important factor in species definition (Stubbe, 1989; Dietrich et al., 1997).

## GENERAL CULTIVATION SCHEME IN THE GREENHOUSE

*Oenothera* is a robust plant and can be grown easily in the greenhouse or in field plots in temperate regions, although not all taxa are equally well suited for both venues. Under optimal cultivation conditions, the generation time of *Oenothera* comprises 4*–*6 months, depending on the strain. Cultivation times range from around 4 months for the strains of the subsection *Munzia* and 6 months for species of subsection *Oenothera*. However, several important laboratory strains in subsection *Oenothera* complete their life cycle in 5 months.

Long-day conditions (16 h light/8 h dark) are required in all stages of a greenhouse culture, except for vernalization (see below). With the exception of *Oe. grandiflora*, short-day conditions inhibit flower formation (see below). Standard growth temperatures range from 18 to 22°C. Higher temperatures such as 24°C (Glick and Sears, 1994; Johnson et al., 2009b) are tolerated and can even promote growth. They should, however, not exceed 27°C since for example bolting may be prevented (Yaniv et al., 1989; Clough et al., 2001; Giménez et al., 2013; but also see von Arx et al., 2012). Plants start to develop the first true leaves 7*–*10 days after germination at a plant size of 2.0*–*2.5 cm diameter. At this stage, they are transferred from seedling trays to individual pots. About 3*–*4 weeks after germination, plants reach the early rosette stage with a diameter of about 12 cm and develop the 5th and 6th leaves (**Figures 1A,B**). Depending on the line, the stage corresponds approximately to stage 1.5 in the *Oenothera* developmental code developed by Simpson (1994). This is the earliest stage, in which plants can be vernalized and transplanted into the final pots of 18 cm for greenhouse cultivation or alternatively into field plots (see below). However, also bigger and stronger plants can be used for transplanting in field cultures. Hence, vernalization and transplanting at later stages is possible, but plants should not be kept too long in 6 cm pots, since they become pot-bound or crowd each other. After transplanting, a burst of growth is observed. Depending on the strain/species, bolting starts 6*–*8 weeks and flowering 10*–*15 weeks after germination (cf., de Vries, 1913; **Figures 1C–F**). Subsection *Munzia* develops faster. With the exception of *Oe. grandiflora*, which bolts very earlier and but might need special treatments for flower induction (see below), flowering always follows bolting. Seed ripening requires another four (subsection *Munzia*) to eight weeks. Fresh seeds can be sown immediately after harvest (cf., Steiner, 1968; Baskin and Baskin, 1994). In the field, the plant dies after completing its life cycle (Hall et al., 1988; Dietrich et al., 1997). However, also iteroparous behavior, i.e., flowering in two subsequent field seasons has been reported, especially for sandy habitats with moderate plant density (Johnson, 2007). In general, production of successive flowers depends on day-length and terminates in short-day conditions, i.e., at the end of a field season but not in long-day greenhouse conditions. For growth schemes of *Oenothera* cultures under various conditions see, e.g., Kachi and Hirose (1983); Gross and Kromer (1986), Russell (1988); Reekie and Reekie (1991), Fieldsend and Morison (2000b); Clough et al. (2001), Deng et al. (2001); Fieldsend (2004), Honermeier et al. (2005), Vilela et al. (2008), or Giménez et al. (2013).

## SUBSTRATE TYPES AND IRRIGATION

*Oenothera* can be grown on a range of substrates. However, as a ruderal, calcicoles weed occurring on sandy or gravelly soils (Hall et al., 1988; Dietrich et al., 1997), it prefers well-drained substrates, tolerates low nutrient contents (cf., de Vries, 1913) and moderate watering. Nevertheless, the plant has a high water demand during bolting, when it grows rapidly. To achieve optimal results, plants must be fertilized. In ecological field studies, the largest biomass and seed production were observed in tilled fields with rich soils (Johnson and Agrawal, 2005; Johnson, 2007; Johnson et al., 2008).

At the Max Planck Institute in Golm, seeds were germinated on and transferred to peat-based commercial substrates optimized for *Arabidopsis* growth (e.g., MPG-mixture from Stender AG, Luckenwalde, Germany) that contain fibric peat, vermiculite, and sand [7:2:1], supplied with 150 mg/l microelements, 100 mg/l Fe-chelate and 1 g/l of the slow-release NPK-fertilizer “Osmocote Start” (Everris International B.V., The Netherlands). Older plants were transferred to “*Oenothera* substrate,” a mixture of the peat-based substrate “Standard Potting Soil Classic,” quartz sand, and fine-grained vermiculite [4:2:1], supplied with 3 g/l “Osmocote Exact Standard 3–4 M” (Everris International B.V., The Netherlands). “Standard Potting Soil Classic” is a soil mixture supplied by the company Einheitserde- und Humuswerke Gebrüder Patzer GmbH & Co.KG, Sinntal/Altengronau, Germany. It is composed of natural clay, fibric and sod peat, supplemented with 250*–*450 mg/l nitrogen, 250–450 mg/l P_2_O_2_, as well as K_2_O (360–500 mg/l) at pH 6.0 (Köhl, unpublished; **Table 1**).

**Table 1:** Cultivation conditions for *Oenothera* in climate controlled glasshouses or foil greenhouses without heating/cooling.

Working step	Controlled environment	Foil greenhouse
**Sowing**
Container (internal dimension)	Seed flat (50 cm × 32 cm, depth 6 cm)	Storage container with perforated bottom (35.5 cm × 25.5 cm, depth 10.5 cm)
Substrate	Stender MPG-mixture for *Arabidopsis thaliana*^1^	Stender MPG-mixture for *Arabidopsis thaliana*^1^
Fertilizer	None	None
Plants per container	50–100 seeds	50–100 seeds
**Transferring**
Time (das)^2^	14–21	14–21
Container (dimension)	Round 6 cm pot	Round 10 cm pot
Substrate	Stender MPG-mixture for *Arabidopsis thaliana*^1^	*Oenothera* substrate^3^
Fertilizer	1 g Osmocote start/l substrate	3 g/l Osmocote Exact Standard 3–4 M
Plants per container	1	1
		
**Planting**
Time (das)^2^	40–50	40–50
Container (dimension)	Round 18-cm pots (diameter 17.5 cm, height 16.5 cm)	Round 18-cm pots (diameter 17.5 cm, height 16.5 cm)
Substrate	*Oenothera* substrate^3^	*Oenothera* substrate^3^
Fertilizer	3 g/l Osmocote Exact Standard 3–4 M	3 g/l Osmocote Exact Standard 3–4 M
Plants per container	1	1

1 Composition of Stender MPG-mixture: fibric peat, vermiculite, and sand [7:2:1], supplied with 150 mg/l microelements, 100 mg/l Fe-chelate.

2 Days after sowing.

3 Composition of *Oenothera* substrate: peat-based substrate “Standard Potting Soil Classic” (natural clay, fibric and sod peat, supplemented with 250–450 mg/l nitrogen, 250–450 mg/l P_2_O_2_, as well as K_2_O 360–500 mg/l at pH 6.0), quartz sand, and fine-grained vermiculite [4:2:1]). Before sowing or transferring, the substrate is soaked with tap water containing 0.906 mg/l Propamocarb to prevent damping off.

In the laboratory of Wilfried Stubbe, Peat Culture Substrate 1 was used up to the early rosette stage and Peat Culture Substrate 2, a soil mix with higher fertilizer concentration, for successive cultivation (Linne von Berg, 1990). Substrates used by the laboratory of Marc T. J. Johnson include Sunshine Mix 1 or Pro Mix General Purpose Peat Soil with vermiculate and a balanced Osmocote [13:13:13], sometimes with micronutrients (e.g., Johnson et al., 2009b).

## GERMINATION

Germination of *Oenothera *seeds requires light and moisture. Seeds are therefore sown on the substrate surface and covered with a translucent dome to maintain humidity. Seedlings appear after one or two weeks. With this method, seed quality, age and/or strain can affect germination capacity and rate substantially up to the point of extreme delays. Large difference can be observed even between different sowings of the same seed lot (Davis, 1915; de Vries, 1915). Germination rates and synchronization can be increased by soaking seeds for 24 h at low temperatures (4*–*10°C) in the dark (cf., Ensminger and Ikuma, 1988). Seeds soaked in 0.1% Agarose can be spread more easily with a pipette. Reliable complete germination can be achieved by transferring imbibed seeds to continuous light and 28°C (cf., Gross, 1985; Ensminger and Ikuma, 1987a).

**FIGURE 2:**
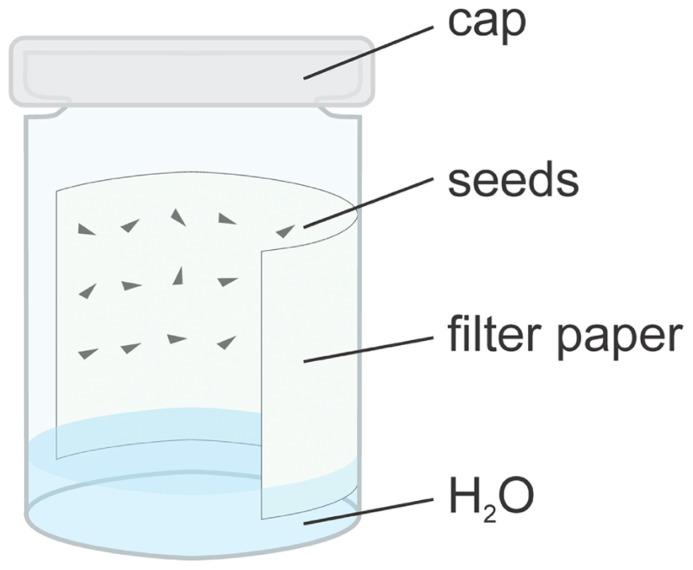
**Germination of *Oenothera* seeds in a humid chamber.** For details see text.

Since such conditions are difficult to achieve in the greenhouse, seeds should be germinated on wet blotting paper in a petri dish (cf., de Vries, 1913; Davis, 1915; Renner, 1917). More efficiently, the seeds can be placed on a wet filter paper, which is placed on the wall of a glass beaker or vial with a lid. The moisture of the paper is maintained by covering the bottom of the beaker with tap water and dipping the filter paper into it (**Figure 2**). In this system, seeds germinate completely within 1*–*3 days (de Vries, 1915, 1916, 1917). If germination is delayed, fungi may appear on the seeds and filter paper, which requires rinsing seeds with a 3% hydrogen peroxide solution (Linne von Berg, 1990). Low quality seed lots from sub-optimal storage or of increased age (see below) require surface-sterilization with hypochlorite. These seeds can be sown on sterile filter paper (Zupok, unpublished) or on sucrose free ½ MS medium (cf., Chiu et al., 1988; de Gyves et al., 2001). Delayed seed germination can be overcome by pressing water into soaked seeds with 6*–*8 bar for 2*–*3 days (de Vries, 1915), or by repeated incubation at 10*–*15°C for the same timeframe. Subsequently, seeds must be returned to higher temperatures (Renner, 1917). Likewise, wetting/drying cycles, stratification, and exposure to hot temperatures can break dormancy. In commercial cultures, soaking at 45*–*50°C for 24*–*48 h, or early spring burial in frozen soils is sometimes applied to promote germination. Other methods, to improve germination rates such as application of sulfuric acid, abrasion, or priming, do not give satisfactory results (for references, see de Vries, 1915; Steiner, 1968; Hall et al., 1988; Nightingale and Baker, 1995; Deng et al., 2001; Wees, 2004; Xu et al., 2010).

Interestingly, light quality has an influence on germination (Gross, 1985). Best germination results are obtained in the spring, when seeds are exposed to sunlight (cf., Davis, 1915; de Vries, 1917; Linne von Berg, 1990; Johnson and Agrawal, 2005). In general, *Oenothera* seeds display a seasonal change of dormancy. Germination is regulated by a subtle interplay of temperature and the duration of exposure to a particular temperature, light quality, quantity, time point and length, storage conditions and length, or date of harvest. Also genotypic differences have been recognized. Under certain conditions, light is not even required for germination (Davis, 1915; Fujii and Isikawa, 1961; Schwemmle, 1961; Steiner, 1968; Gross, 1985; Ensminger and Ikuma, 1987a,b; Ensminger and Ikuma, 1988; Baskin and Baskin, 1994; Doroszewski, 2007).

Interesting exceptions from the standard germination protocols are species with fruits that do not dehisce (i.e., section *Gaura*). Capsules, which only contain one to four (five) seeds (Wagner et al., 2013), are placed in 1 cm depth into the substrate and exposed to fluorescent white light at regular greenhouse temperatures until seedlings establish. This procedure is required because the seeds are embedded in very hard tissue. 1*–*2 days of soaking the capsules does help to loosen the carpels, but it is still insufficient to extract the seeds effectively. In soil, the tissue, in which the seeds are embedded, decomposes quickly after a few days (Johnson, unpublished; Hollister et al., submitted).

Germination can be influenced by seed infection with *Septoria oenotherae*, an Ascomycota species developing pycnidia (asexual fruit bodies) within seeds. The pathogen is ubiquitously present in *Oenothera* cultures (Simpson et al., 1995; O’Connell et al., 2005). Heavily infected seed lots exhibit reduced or no germination. Adding 0.05% of Plant Preservative Mixture (Plant Cell Technology Store, Washington, DC, USA) to germination media in tissue culture plates, or 3% of the chemical while soaking the seeds, significantly enhances germination rates (Stegemann et al., unpublished). Incubation of the seeds in 45°C hot water for 25 min destroys the fungus without affecting germination (O’Connell et al., 2005). Long-term steed storage at –20°C (see below) reduces fungal survival (Greiner, unpublished). Finally, it should be mentioned that in some permanent translocation heterozygous strains 50% of the seeds are aborted as a consequence of sporophytic lethal factors (cf., Cleland, 1972).

## BOLTING AND FLOWER INDUCTION

Requirements for flower-induction are quite diverse within the whole genus *Oenothera*, and vernalization response is not uniform within a species. Depending on the material, various kinds of behaviors have been reported. For example, most of the strains of subsection *Munzia* do not require vernalization when grown in long-day conditions. *Oe. grandiflora* is described as a short-day species, but flowers under long-day conditions in the greenhouse, but not in the field (Steiner and Stubbe, 1984; Greiner, unpublished; Johnson, unpublished; see below). Some lines can be devernalized in short day (and need long days after vernalization), whereas others are day neutral after vernalization (for reviews and references see Chouard, 1960; Wellensiek, 1965; Steiner and Stubbe, 1984; Takimoto, 1985; Clough et al., 2001; Wilkins and Anderson, 2007).

Many *Oenothera* taxa, however, are winter annuals or (facultative) biennials (Dietrich, 1977; Hall et al., 1988; Dietrich et al., 1997). In these genotypes, flower induction in the greenhouse requires vernalization followed by a long-day exposure. Alternating cold–warm treatments are more effective than continuous cold (Chouard, 1960; Picard, 1965). Seed vernalization seems to be ineffective (Chouard, 1960; Picard, 1965; but also see Thomas and Vince-Puce, 1997, p. 161; Deng et al., 2001), but some vernalization response was observed in seedlings by Giménez et al. (2013). In some materials, predisposition for vernalization starts 30 days after germination, which corresponds to the end of the early rosette stage described earlier, peaks after 56 and again after 180 days (Müller-Stoll and Hartmann, 1959; reviewed in Chouard, 1960; Takimoto, 1985). Ecological studies indicate that effective induction of bolting requires a minimal rosette diameter of 9*–*12 cm (Gross, 1981; Kachi and Hirose, 1983; but also see Giménez et al., 2013 above). However, according to field observation by Marc T. J. Johnson, rosette size is not strictly correlated with bolting capability. In stressful environments, the minimal rosette sizes which allow shoot induction can be very small, but can reach more than 50 cm in productive environments.

Although the photoperiod during vernalization seems be unimportant (cf., Picard, 1965), *Oenothera* is typically vernalized for 7*–*14 days at 4*–*8°C in a 10 h light/14 h dark cycle (cf., Clough et al., 2001). After vernalization, plants are transferred to large pots and returned to standard greenhouse conditions (long day, 16 h light/8 h dark). Unfortunately, some lines of *Oe. biennis*, *Oe. villosa*, and probably some *Munzia* strains, bolt erratically after this treatment. In a typical representative of this group, a line of the true European biennis (cf., Renner, 1942; Rostański, 1985), flower induction required an 11°C day/3°C night treatment for more than 10 weeks, followed by a photoperiod longer than 12 h (e.g., Picard, 1965; for review see Chouard, 1960; Takimoto, 1985). We found that repeated, prolonged and harsh vernalization cycles together with transplanting into fresh substrate can induce flowering in such lines. However, these treatments are unreliable and prolong cultivation time significantly (Greiner, unpublished). Spray application of gibberellic acid generally induces bolting and subsequent flowering in most Oenotheras (Chouard, 1960; Takimoto, 1985; Reekie and Reekie, 1991; Greiner, unpublished). However, the “bolting resistant” line studied by Chouard and co-workers only responded by stem elongation and lignification, but neither bolted nor flowered (Chouard, 1960; Picard, 1965, 1967). The best alternative for the “bolting resistant” materials is hence to plant them into field no later than early May, when (artificial) vernalization is no longer required. This treatment is obligate and done so, the plants flower reliably (Renner, 1948; Chouard, 1960; and see below).

In summary, vernalization requirements for *Oenothera* can roughly be grouped into three categories: (i) strains/species with no or only very moderate demand for vernalization, (ii) material with “normal” vernalization requirements, and (iii) “bolting resistant” lines. Which strain belongs to which group is to some extent independent from the species and the optimal treatment for each line must be determined experimentally. Vernalization or planting to the field early in May does not harm genotypes without a vernalization requirement and is therefore recommended as standard operating procedure for genotypically diverse cultures.

## CROSS-POLLINATION AND SELFING, SEED HARVEST, PROCESSING, AND STORAGE

Especially in section *Oenothera*, seed numbers per capsule are quite variable ranging from 180 to 500 (Hall et al., 1988; Harte, 1994; Dietrich et al., 1997). The seed yield per capsule is highest on the main stem, and also depends on the position at the stem. The first three to four flowers of a shoot produce significantly fewer seeds, and lower yields are also observed for the last 5*–*20 capsules (Stubbe, unpublished; Greiner, unpublished; Johnson, unpublished). For selfing of small-flowered, permanent translocation heterozygous strains, it is sufficient of remove the shoot tip and bag the remaining inflorescence. In those lines, if not anyway cleistogamous, anthers overgrow the stigma, which ensures pollination. Large-flowered, outcrossing, bivalent-forming lines, where anthers are shorter than the style, benefit from additional hand-pollination. For cross-pollination, flowers with closed microsporangia should be emasculated 24 h before flower opening. Such flowers no longer display “vitreous” petals. Cleistogamous lines must be emasculated earlier. Typically, one stem displays three to five flowers in the suitable developmental stage. The remaining flowers below and flower buds above are removed. Cross-pollination is conducted when the flower is open, typically the following day. Direct pollination after emasculation is possible, but reduces yield, especially in cleistogamous material. The best pollen is obtained from flowers whose anthers already have released their pollen, one or half a day before flower opening. Since in *Oenothera* pollen grains are connected by viscin threads, special care must be taken to avoid unintentional pollen transfer. Cleaning tweezers and hands with 70% EtOH and removing pollen threads from flowers before emasculation by washing or spraying with water are effective methods. Mature pollen frozen in liquid nitrogen and stored at –80°C can remain fertile for at least three years, although this has not yet been investigated systematically, and fertilization success using such pollen has been variable so far (Greiner, unpublished). For bagging of inflorescences, we use pergamin bags (HERA Papierverarbeitung Puttrich GmbH & Co KG, Germany, Cat. No. 720P40), that withstand rainfall and are thus well-suited for field experiments (cf., de Vries, 1913). Wet bags have to be exchanged soon after rainfall, to prevent mold from growing on the inflorescences. A third to half of the bag volume should be left empty, since stems will still grow by cell elongation after tip removal. Since lateral stems have a tendency to break from the rosette, especially in lines containing a C-genome, they must be tied to poles. When labels with a long wire are used (Hermann Meyer KG, Germany, Cat. No. 110130), bag closure, tying, and labeling can be conducted in a single step (cf., de Vries, 1913; and methods of the laboratories of Wilfried Stubbe, Reinhold G. Herrmann and Stephan Greiner).

The recognition of the best harvest time is not trivial. Ripe capsules turn brown and split. Among the alternating capsules on a stem, the basal ones can already disperse their seeds while the upper ones are still unripe. This makes an efficient harvest challenging, particularly in selfed plants with many capsules on a stems. Especially in the subsection *Munzia*, where shattering can be extreme, plants must be checked daily for ripe capsules during harvest. In addition, there is even a strong proximal/distal ripening gradient within the capsules of this subsection. Stems with ripe capsules can be dipped into 95% EtOH and flamed before drying to reduce fungal contamination during subsequently germination (Sears and Klomparens, 1989; Sears, unpublished). Seeds must be dried for at least six weeks before processing for long-term storage. This can be done at room temperature between paperboards, or, in climate chambers at 15°C and 15% relative humidity. Subsequently, seeds should be frozen at –20°C. Seeds stored at room temperature fail to germinate after 3*–*5 years (cf., de Vries, 1916). Storage at lower temperature (4°C) prolongs seed life span significantly (cf., de Vries, 1913). A critical aspect of long-term seed storage is seed moisture. Seeds dried to 5% residual moisture (1 month in a dessicator with CaCl_2_ at room temperature) and stored in hermetically sealed packages (Dietrich, unpublished) remain viable for more than 25 years (Greiner, unpublished). Seeds buried in moist soil can remain viable for 80 years (Telewski and Zeevaart, 2002). Seed lifetime also depends on the nutrition status and vigor of the mother plant (de Vries, 1913).

## PEST CONTROL

There is a general agreement in the literature about an enhanced resistance of *Oenothera* against diseases or pests (e.g., de Vries, 1913; Fieldsend and Morison, 1999; Deng et al., 2001). However, various insect pests, fungal and even intracellular bacterial pathogens were reported, which infect wild and cultivated evening primroses, including roots and seeds (e.g., de Vries, 1913; Hall et al., 1988; Sears and Klomparens, 1989; Venkatasubbaiah et al., 1991; Simpson et al., 1995; Deng et al., 2001; Johnson and Agrawal, 2005; Rakauskas, 2008; Agrawal et al., 2012; and references therein). Our experimental cultures of *Oenothera*, grown in Golm (Brandenburg, Germany) or Munich (Bavaria, Germany) were regularly infested with aphids and powdery mildew, more rarely downy mildew, white flies, and sap beetles. Besides that, invasion by scale insects has been reported for North America (Johnson, unpublished). Fungal infections by *Botrytis* occur especially at low temperatures in autumn. Young rosettes, but also flowering plants, are to some extent susceptible to thrips infection. In field cultures, damage by root voles was also observed. Infections by thrips, aphids, as well as downy mildew can quickly spread and cause irreversible damage. In our experience the pesticides mentioned below are widely tolerated by *Oenothera*, although sometimes flower abortion might result from Acetamiprid application (5 mg/m^2^) under certain environmental conditions. For tolerance of further insecticides see Agrawal et al. (2012).

The most suitable method of protection against thrips depends on the exact species that is responsible for the damage. Problematic are typical glasshouse thrips like *Frankliniella occidentalis* or *Thrips tabaci*, since they rapidly develop pesticide resistance and therefore cannot be controlled exclusively by insecticides. The most effective action is the proactive use of predatory mites. *Amblyseius cucumeris* (Katz Bio Tech AG, Germany, Cat. No. 4085) is applied to the leaves and kills the larvae of thrips. Another predator mite, *Hypoaspis miles* (Katz Bio Tech AG, Germany, Cat. No. 4140), is applied to the soil on planting and kills soil-dwelling stages. The biological control will become ineffective if temperatures are higher than 28°C, which then may lead to mass-development of thrips. These can be controlled with Azadirachtin (3 mg/m^2^) or Spinosad (27 mg/m^2^) up to twice per year. Care must be taken because the predators are sensitive to many pesticides. The aphid species that commonly infests *Oenothera* in European greenhouse cultivation is *Myzus persicae*. This species can be controlled with Acetamiprid (5 mg/m^2^), Pirimicarb (17.5 mg/m^2^) or Pymetrozin (18 mg/m^2^). Acetamiprid and Pymetrozin must not be used on flowering Oenotheras that are being visited by bees. Furthermore, similar to greenhouse thrips, *M. persicae *rapidly develops pesticide resistance. Insecticides like rape seed oil or soap solutions that cause less resistance problems did not yield satisfactory result, although *Oenothera* tolerates these substances, if light intensities are less than 600 μmol s^-^^1^ m^-^^2^. Under suitable conditions (day length > 12 h, temperatures > 15°C), preventive biological control with aphid-parasitic *Aphidius *wasps like *Aphidius colemani* (Katz Bio Tech AG, Germany, Cat. No. 4050) should be applied. In North America, the situation can be quite different. Here, specialized aphids, including *Macrosiphum gaurae* and *Aphis gaurae* can attack *Oenothera* plants (Johnson, unpublished). White flies (Aleyrodidae) infect *Oenothera* species in the greenhouses without insect nets. As the chemical control of white flies is ineffective, preventive biological control with *Encarsia formosa* (Katz Bio Tech AG, Germany, Cat. No. 3050) needs to be implemented at the beginning of the season.

Fungal infection of *Oenothera* with *Botrytis cinerea*, *Pythium*, or downy mildew (Peronosporaceae) is best prevented by hygiene measures in the greenhouse, e.g., regular removal of dead plant material and cleaning of all surfaces including recycled pots with soap and water or a benzoic acid-based greenhouse disinfectant. Substrates for sowing or transferring *Oenothera* seedlings can be pretreated with Propamocarb (0.906 mg/l) to prevent damping off (**Table 1**). Downy mildew infections can be prevented by avoiding wet leaves, and controlled by Azoxystrobin (25 mg/m^2^). Powdery mildew (Erysiphaceae), in contrast, thrives at low air humidity and on older plant material. The infection rapidly spreads from older to younger plants when both are grown in the same greenhouse. Fungicides containing Tolylfluanid (390 mg/m^2^) or Azoxystrobin (25 mg/m^2^) kill powdery mildew but do not prevent reinfection. Powdery mildew rapidly becomes resistant to various fungicides. Application of sulfur spray was found to be ineffective and interferes with the biological thrips and aphid control (methods for *Oenothera* developed by Karin Köhl).

For further references on chemical pest control for various diseases, see, for example, Grignac (1988), Reeleder et al. (1996), Murphy et al. (2004), Johnson et al. (2009a), Agrawal et al. (2012), or Giménez et al. (2013). Many older pesticides are no longer registered and thus must not be used in greenhouses or fields, however. To obtain uninfected cultures in experimental fields, early clearing of the field in autumn and plot rotation is of particular importance (cf., de Vries, 1913; Reeleder, 1994; O’Connell et al., 2005).

Interestingly, some *Oenothera* strains homozygous for the A-genome, display a remarkable resistance against the major pests in experimental cultures, aphids and powdery mildew. This is even the case under very high infection pressures. On the other hand, strains containing a B-genome, as well as *Munzia* lines, are comparably susceptible to aphid or powdery mildew infections. Within the two groups genotypic variation is observed (Hall et al., 1988; Johnson and Agrawal, 2005; Agrawal et al., 2012; Hersch-Green et al., 2012; Greiner, unpublished).

*Oenothera* displays broad resistance to pre- and post-emergence herbicides (Stringer et al., 1985; Richardson and West, 1986; Bates et al., 2013a,b). This might be utilized for effective weed control, especially in agronomics (Grignac, 1988; Roy et al., 1994; Honermeier et al., 2005; Ghasemnezhad and Honermeier, 2007). However, reports from the genetic literature are lacking so far.****

## CULTIVATION IN FOIL GREENHOUSES AND EXPERIMENTAL FIELDS

In the temperate climate zone of the northern hemisphere, *Oenothera* seeds are traditionally sown in January, seedlings are selected and potted in February, and transplanted to the field in April or May (e.g., de Vries, 1913; Gates, 1914; Schwemmle et al., 1938; Renner, 1948). Plants pre-cultivated in warm greenhouses that exclude ultraviolet radiation need to be hardened at the beginning of April, e.g., in a cold-frame with a shade-cloth, before they are transplanted to the field in the beginning of May (Linne von Berg, 1990). Requirement of fertilization depends on the nutrient content of the soil, which ought to be determined before plants are transplanted. The average planting distance is 30*–*35 cm (de Vries, 1913). In spite of its size, *Oenothera* is not very competitive in experimental field cultures (cf., Gross and Werner, 1982; but also see Johnson et al., 2008; Turley et al., 2013). Thus, weeds need to be controlled either manually or with a selective herbicide (see above). Plants start flowering in July and August. Crossings and self-pollination should be performed within six weeks (de Vries, 1913; Renner, 1917; Steiner and Stubbe, 1984). Seeds are harvested in September or October. Plants that flower later than the beginning of September rarely produce mature seeds, unless grown at lower latitudes. Non-bolted plants can be overwintered, although this may transfer pests and diseases to next year’s field plots (de Vries, 1913; Johnson, 2007; and see above).

The schedule outlined above allows synchronization, flower induction, and seed maturation in large experiments with diverse genotypes within a growing season. Modern greenhouse facilities enable the compression of this schedule and allow sowing in March. Still the material must be planted into the field in the beginning of May, that it can undergo natural vernalization (Renner, 1948; Roy et al., 1994; but also see Johnson, 2007). Genotypically diverse cultures planted in June obligatorily require vernalization, but this still runs the risk that the “bolting resistant” lines fail to flower (see above). Material planted in June should be grown in containers to facilitate the transfer into the greenhouse in October/November for final seed maturation. Re-routing of field grown material or grafting of unripe stems regularly fails (Greiner, unpublished). However, Rossmann (1963) reports 90% ripening success by placing half ripe stems in Knops’s or Crone’s nutrient solution.

As an alternative to field cultivation, *Oenothera* can be grown in pots in unheated polyethylene foil greenhouses (cf., **Table 1**). These greenhouses are substantially cheaper than glasshouses and prolong the season by 2*–*3 months, by allowing a 4*–*6 week earlier start of the growth period and up to 2 months extension at the end of the season. Within the greenhouse, plants can be grown in pots, with minimal weeding. Plants are protected from the weather that can severely damage some “fragile” lines in the field. Rain protection makes the exchange of wet pollination bags unnecessary.

In both, the field and polyethylene foil greenhouses, additional treatments are required to induce flowering of *Oe. grandiflora*. The species has the ability to flower in long days when grown under standard greenhouse conditions (see above). However, when grown in field or in foil greenhouses in the North (e.g., Ann Arbour, MI, USA; Düsseldorf/Munich/Potsdam-Golm, Germany), *Oe. grandiflora* sets flower buds only as late as September, when plants experience the natural transition to short day (Steiner and Stubbe, 1984; Greiner, unpublished). *Oe. grandiflora* lines can be synchronized with other strains by a short-day regime of 10 h light/14 h dark, which is started when plants start bolting in long days and finished when flower buds are formed. Plants can then be planted in the field and will bloom in July and August (Steiner and Stubbe, 1984; Linne von Berg, 1990). However, such a treatment requires special growing facilities. Hence, alternatively one might grow *Oe. grandiflora* in a climate-controlled greenhouse in long days, in parallel to the other strains in the field/foil greenhouse. Another possibility for synchronization is to delay the complete field or foil greenhouse cultivation by late planting in June. However, special care must be taken to induce bolting of the strains not belonging to *Oe. grandiflora* and, most importantly, to allow adequate time for seed ripening late in the season (see above). In addition, *Oe. grandiflora* strains that bloom early in the field were reported (Steiner and Stubbe, 1984), which could be selected to simplify the crossing of multiple lines.

## AGRICULTURAL CULTIVATION

Cultivars of subsection *Oenothera* are commercially grown as an oil seed crop for the production of gamma-linolenic acid (Russell, 1988; Simpson and Fieldsend, 1993; Deng et al., 2001; Ghasemnezhad and Honermeier, 2007). Due to their faster life cycle, some species in the subsection *Munzia* have been tested. These, however, displayed insufficient gamma-linoleic acid levels (Vilela et al., 2008).

A substantial amount of literature dealing with *Oenothera* crop management has accumulated, and a comprehensive review would be beyond the scope of this article. In brief, *Oenothera* is grown in temperate climates as winter and spring crop (Grignac, 1988; Simpson and Fieldsend, 1993; Deng et al., 2001). Spring cultivars, depending on the regional climate, can be drilled until mid-April. Plants start flowering in August and are harvested in October. Winter crops are started in August, overwinter as rosettes, flower in July and produce ripe seeds in September (Fieldsend and Morison, 1999; Fieldsend and Morison, 2000b; Honermeier et al., 2005). Winter crops require more effort in crop management, but allow an earlier harvest (Fieldsend and Morison, 2000a,b; O’Connell et al., 2005). In terms of yield or oil quality, no clear recommendation for spring and winter crops can be given. Among the investigated cultivars, spring crops tend to produce oil with a higher percentage of gamma-linoleic acid. Better overall oil content can be observed in winter-grown material, which has a higher biomass and potentially greater seed yields (Fieldsend and Morison, 1999; Fieldsend and Morison, 2000a,b; Honermeier et al., 2005). Nitrogen fertilization has minor effects on the yield and in any case should be applied moderately (Russell, 1988; Stobart and Simpson, 1997; Şekeroǧlu and Özgüven, 2006; Ghasemnezhad and Honermeier, 2008). The most important factors for seed/oil yield and quality are harvesting time and method, as well as seasonal variation of environmental factors, e.g., growth temperature (Yaniv and Perl, 1987; Yaniv et al., 1989; Levy et al., 1993; Simpson and Fieldsend, 1993; Fieldsend and Morison, 2000a; Ghasemnezhad and Honermeier, 2007). Breeding goals for *Oenothera* include non-splitting capsules, high seed and oil yields, or high gamma-linoleic acid content. All of these were successfully combined, e.g., in the cultivar Rigel (Fieldsend, 2007).

## INTERSPECIFIC CROSSES, CROSS FERTILITY, AND FERTILITY OF HYBRID OFFSPRING

Self-incompatibility is found to some extent in the entire genus *Oenothera* (Wagner et al., 2007), and was reported already very early, e.g., from *Oe. organensis* (subsection *Emersonia*; Emerson, 1938). However, self-incompatibility is less important in the genetically most relevant subsections. Self-incompatibility alleles have not been found in subsection *Munzia* (Dietrich, 1977) and they are rare in subsection *Oenothera*. Here, they occur in *Oe. grandiflora* (Stubbe and Raven, 1979b) and, according to work of Erich E. Steiner, in some lines of *Oe. biennis*. In this species they stabilize permanent translocation heterozygosity (for review see Cleland, 1972). As a rule, within the subsections of section *Oenothera*, species can be hybridized freely, but offspring often express plastome–genome incompatibility. Besides chlorosis, these hybrids usually develop normally, but occasionally, sterility occurs or particular plastome–genome combinations result in severe growth retardation (e.g., Schwemmle et al., 1938; Schwemmle and Zintl, 1939; Stubbe, 1963; Cleland, 1972; Stubbe et al., 1978; Stubbe and Raven, 1979a; Stubbe, 1989; Harte, 1994; Stubbe and Steiner, 1999; and references therein). Hybrid incompatibility conferred by nuclear alleles is sometimes found between crosses of particular strains (e.g., Renner, 1917; Rossmann, 1963; Jean, 1984; Greiner, unpublished). In contrast to plastome–genome incompatibilities, these nuclear hybrid incompatibilities result from the genetic composition of the particular hybrid, which is often not connected with the taxonomic positions of the parents. Similar results are obtained, if species between the subsections of section *Oenothera* are hybridized. Although such hybrids are more difficult to obtain, and sometimes seed abortion is observed, inter-subsectional crosses generally produce offspring. The hypanthium length and/or the plastid (chloroplast) genotype are the most important, but not exclusive, barriers. Plastid genotype and hypanthium length facilitate realization of certain hybrids only in one crossing direction, i.e., the maternal parent must have a short hypanthium, allowing the paternal pollen tube to reach the egg cell, and/or must have a compatible plastid genotype in order for embryos to be able to develop. Other consequences of plastome–genome incompatibility include severe growth retardation, sterility or reduced fertility and/or meiotic irregularities in the resulting hybrids. To obtain particular hybrids, sometimes at least one parent must be equipped with a certain plastome type (Stubbe and Raven, 1979a). On the other hand, complete pollen sterility or seed abortion might be observed as a consequence of cytoplasmic exchanges between subsections (e.g., Kistner, 1955; Arnold, 1970). For references on the rich literature of inter-subsectional crosses between sections *Oenothera* and *Munzia*, see Stubbe and Raven (1979a).

## VEGETATIVE PROPAGATION, INDUCTION OF POLYPLOIDY, AND MODIFIABILITY

Vegetative propagation by stem cuttings is reported from ornamental strains of *Oe. fruticosa* (section *Kneiffia*; Clough et al., 2001). It is, however, rather ineffective in the subsection *Munzia* and *Oenothera*. In subsection *Oenothera*, new rosettes can be propagated from lateral rosette buds (de Vries, 1913). In addition, plants occasionally produce side rosettes. The signals triggering the production of such rosettes remain unclear (Greiner, unpublished). Also, young rosettes can be split and planted directly into moist soil. These plants will develop full rosettes again. Alternatively, they can be re-rooted in water or in a moist chamber first (Golczyk and Greiner, unpublished). Interestingly, some species within section *Oenothera* (especially *Oe. humifusa* and *Oe. drummondii*) often display root induction from a cut leaf surface, when leaf pieces are placed on wet filter paper for 1–2 weeks (Johnson, unpublished).

Species of subsections *Oenothera* and *Munzia* are exclusively diploid (Cleland, 1972; Dietrich, 1977; Dietrich et al., 1997), but effective protocols for the production of polyploid lines are available. Seeds or seedlings are placed on wet blotting paper containing colchicine, or alternatively rosettes are treated with colchicine shortly before bolting. In both cases, most surviving plants produce tetraploid offspring (Hecht, 1942; Stomps, 1942; Linnert, 1950; Renner and Hirmer, 1956). Subsequent treatments of tetraploid lines with colchicine can produce octo- or even hexadecaploid plants (Renner and Hirmer, 1956; Rossmann, unpublished). For non-permanent translocation heterozygotes, haploid lines are reported with a spontaneous frequency of 0.1*–*0.2% (Davis and Kulkarni, 1930; Harte, 1973). Additionally they can be induced by X-ray treatment (Linnert, 1950; Harte, 1972), or by performing wide interspecific crosses (e.g., Gates, 1929; Steiner, 1952; Schwemmle and Simson, 1956; Lee and Hecht, 1975; Harte, 1994; and references therein). Haploid material can be converted to double-haploid lines by colchicination (e.g., Oehlkers, 1940; Linnert, 1950; Lee and Hecht, 1975).

Plant habits and growth can be modified by abiotic factors. For example, if *Oe. grandiflora* is kept in small pots, plants stay substantially smaller and stress induces flowering extremely early. However, genotypic variation exists (Johnson, unpublished; Greiner, unpublished). A similar effect is observed, in *Oe. elata*, when small rosettes are treated with gibberellic acid (Greiner, unpublished). Application of uniconazole in *Oe. fruticosa *leads to flower induction in comparably small plants, whereas other growth regulators had no effect in this material (Clough et al., 2001).

Cultivation in constant light promotes flowering in plants of subsection *Oenothera* similar to long-day treatment, but can harm some genotypes. Grown in short days, however, plants produce more rosette leaves, which are smaller, but thicker and broader relative to long-day plants. Another contrast is that long-day rosettes have erect leaves, while short-day rosettes lay flat on the ground in a disk-like habit. Low temperatures promote this phenotype. Under such conditions, plants can be maintained for years in the greenhouse, since bolting is prevented. If rosettes, which failed to bolt after vernalization and long-day treatment, are maintained in a greenhouse under standard growing conditions (18*–*22°C, long day), the plants will continue to produce new rosette leaves, but their stems will elongate and thicken, leading to a perched rosette after senescence of older leaves (Renner, 1948; Greiner, unpublished). This reassembles the phenotype obtained from application of gibberellic acid to the “bolting resistant” lines described above. For picture see (Picard, 1965, p. 262).

## GERMPLASM RESOURCES

The laboratory of Stephan Greiner preserves a unique collection of *Oenothera* germplasms consisting of about 1000 accessions. The material mainly, but not exclusively, covers subsections *Oenothera* and *Munzia*, including wild races and laboratory stains. About 350 lines have been analyzed genetically in detail. Among them are hybrids of defined nuclear and plastid composition, chromosome translocation or plastome mutants. The collection was originally set up by Werner Dietrich and Wilfried Stubbe, later harbored by Reinhold G. Herrmann, and is extended since then. It represents most of the genetic work of Hugo de Vries, Otto Renner, Ralph E. Cleland, Wilfried Stubbe, Franz Schötz, Erich E. Steiner, Julius Schwemmle, Erick Haustein, Carl-Gerold Arnold, Adolph Hecht, Friedrich Oehlkers, Günther Rossmann, Reinhold G. Herrmann, Barbara B. Sears, and other *Oenothera* scientists, and is the living reference collection for the taxonomy of subsection *Oenothera* by Werner Dietrich, Warren L. Wagner and Peter H. Raven (Dietrich et al., 1997), as well as the *Munzia* taxonomy of Werner Dietrich (Dietrich, 1977). A large and taxonomically diverse germplasm resource was collected by the laboratory of Marc T. J. Johnson (University of Toronto at Mississauga, ON, Canada). It harbors seeds of over 100 species from more than 1000 populations, with a special focus on the genus *Oenothera*, mostly *Oe. biennis*. Further *Oenothera* germplasm is available from the Ornamental Plant Germplasm Center (OPGC) at The Ohio State University (Columbus, OH, USA). The material is searchable by the USDA/ARS National Plant Germplasm System (Germplasm Resources Information Network, GRIN, www.ars-grin.gov). It includes material from Ralph E. Cleland, Wilfried Stubbe, Werner Dietrich, and Cornelia Harte, covering a substantial number of lines from the genetic literature, not only from the afore mentioned laboratories. *Munzia* species, commercial cultivars, and taxa from other *Oenothera* sections are present as well. Some of these lines are described as “historical records,” but material provided to us from OPGC easily germinated. In addition, some groups currently working in the field have notable living collections. The largest reported collection of *Oenothera* germplasm so far was the result of a breeding program conducted by Scotia Pharmaceuticals Ltd., UK, through the end of the 1990s and contained about 2000 accessions of subsection *Oenothera* (Fieldsend, 2007).

## Conflict of Interest Statement

The authors declare that the research was conducted in the absence of any commercial or financial relationships that could be construed as a potential conflict of interest.

## Footnotes

1 Sections and subsections are taxonomic ranks below the genus level, used if appropriated in plant taxonomy.
